# Nutrition and diet in the general U.S. Asian American population: A scoping review protocol

**DOI:** 10.1371/journal.pone.0309219

**Published:** 2024-08-23

**Authors:** Sze Wan Chan, Gregory Laynor, Shahmir H. Ali, Stella S. Yi

**Affiliations:** 1 Department of Population Health, Section for Health Equity, NYU Grossman School of Medicine, New York, New York, United States of America; 2 NYU Health Sciences Library, NYU Grossman School of Medicine, New York, New York, United States of America; UCSF: University of California San Francisco, UNITED STATES OF AMERICA

## Abstract

**Introduction:**

Asian American populations face unique structural/social inequities contributing to poor diet quality and health disparities. The current body of literature on diet and food consumption of Asian Americans mainly focuses on the health of Filipino and East Asian Americans, or those with pre-existing non-communicable diseases.

**Objective:**

The aim of this review is to comprehensively compile all available literature on nutrition and dietary consumption among the general population in Asian American ethnic subgroups, highlight any disparities and research gaps, and suggest further research action.

**Methods:**

With guidance from a research librarian, we enumerated and searched key terms related to diet, food, nutrition, and Asian Americans in PubMed/MEDLINE, Food Science Collection (CABI Digital Library), CINAHL (EBSCO), Scopus, Food Science and Technology Abstracts (Web of Science), and Biological & Agricultural Index Plus (EBSCO) in accordance with PRISMA-S guidelines. An article will be included if it was published in the English language; is a peer-reviewed research manuscript or published in grey literature from 2000 to present; and describes what food groups and macronutrients healthy non-institutionalized Asian Americans in the U.S. are eating. An article will be excluded if it contains only research conducted outside of the U.S.; combines Asian Americans with Native Hawaiian and Pacific Islanders; and had no explicit focus on Asian American nutrition and dietary consumption. Two or more reviewers will participate in the study screening and selection process. We will record article characteristics, diet outcomes, and recommendations from final included articles using a data extraction table and prepare a summary narrative with key findings.

**Expected outputs:**

Results will be disseminated through a peer-reviewed manuscript. The findings from this review can have broad implications for designing and implementing nutrition-focused initiatives that will appropriately reflect and address the needs as well as norms and values of each distinct Asian American ethnic subgroup.

## Introduction

It is known that food plays a significant role in shaping individual and population health and can protect against various non-communicable diseases (NCD), aid in disease management, and support mental health [1]. Yet poor diet quality–contributing to 45% of cardiometabolic deaths and a leading risk factor of attributable disability-adjusted life years in the United States (U.S.) [2]–remains a perplexing public health challenge. According to the Dietary Guidelines for Americans, 2020–2025 (Dietary Guidelines), the core elements of a healthy dietary pattern include consumption of nutrient-dense forms of foods and beverages across all food groups including vegetables of all types, fruits, grains (especially whole grains), low-fat or fat-free dairy, proteins, and oils. The Dietary Guidelines’ recommendation of a healthy dietary pattern is a customizable framework that is tailored to personal, cultural, and traditional preferences [3]. An understanding of cultural and traditional dietary intake and patterns of different population groups is essential to designing nutrition-focused initiatives that will appropriately address health disparities across racial/ethnic groups.

Asian Americans–the fastest growing race/ethnic group in the U.S. [4] expected to reach a national population of 46 million by 2060 [5]–experience significant health disparities related to poor diet including elevated risk of type 2 diabetes (T2D), hypertension, high LDL cholesterol, and low HDL cholesterol [6–13] as well as having one of the highest risks of T2D and non-alcoholic fatty liver disease (NAFLD) of all racial/ethnic minority groups [14–18]. Asian Americans are also the only racial/ethnic group to experience cancer as the leading cause of death. Compared to the general American population, Asian Americans have higher colorectum cancer mortality partially attributable to lifestyle factors including unhealthy diets [19–21]. Yet Asian Americans are the most understudied racial/ethnic group in the United States, with 0.01% of published articles from 1966–2000 listed in MEDLINE including Asian American or Pacific Islanders [22]. The term ‘Asian American’, however, extends beyond the six largest Asian American ethnic groups (Chinese, Indian, Filipino, Vietnamese, Korean and Japanese) that research has traditionally focused on [23]. Given the harms of data aggregation and how it obscures within-group inequities [24, 25], there is a crucial need to broaden inclusion of ethnicities to include a diverse range of origin groups including Pakistani, Thai, Cambodian, Hmong, Laotian, Bangladeshi, Nepalese, Burmese, Indonesian, Sri Lankan, Malaysian, Mongolian, Bhutanese, and other ethnic groups as outlined in previous review papers and the U.S. Census Bureau [23, 26, 27].

In the published literature, Asian American diets are generally characterized by the centrality of white rice, and diet alterations based on acculturation status, such as increased caloric intake, increased consumption of animal protein and dairy/milk products, incorporation of processed snacks/desserts, and decreased consumption of seafood compared to traditional diets [28–38]. But these patterns do not accurately represent the dynamic nature of migration–who is migrating and the globalizing food environments in sending countries. Furthermore, as noted in a recent review on Asian American health research, the nutrition and dietary reviews that were included mainly focused on the health of Filipino and East Asian Americans, or those with pre-existing NCDs such as T2D or hypertension [27]. Little has been published in the literature on the dietary intake and patterns of healthy Asian American populations without NCDs. Also of note is that the majority of existing literature are epidemiological studies that do not describe general diets or sociocultural factors influencing Asian American diets.

Given the gaps in the literature on Asian American diets, this review aims to comprehensively compile all available literature on nutrition and dietary consumption among the general population in Asian American ethnic subgroups, highlight any disparities between groups and research gaps, and suggest further research action. Importantly, scoping out meta-level insights and trends in Asian American eating behaviors is pivotal to inform more tailored policies and health interventions aimed at dietary risk factors behind the emerging Asian American NCD crisis.

## Materials and methods

A scoping review was determined to be more suitable compared to a systematic review because our goals are to broadly understand what has been written about the nutrition and dietary consumption of different Asian American ethnic subgroups [39, 40]. The research team initially considered using the National Institutes of Health (NIH) National Institute on Minority Health and Health Disparities (NIMHD) Nutrition Health Disparities Research Framework [41] to provide a comprehensive and multi-level understanding regarding drivers of Asian American diet at the societal, community, interpersonal, and individual levels. However, given the aim and scope, which is to focus on what Asian Americans are consuming rather than why consumption patterns are the way they are, this review will identify and present available information of diet and food consumption of Asian American ethnic subgroups based on guidelines and key recommendations from Dietary Guidelines for Americans, 2020–2025 [3]. Additionally, while acculturation is an important factor influencing diet [28–31], it falls outside the scope of this review and will not be a focal outcome that will be reported.

### Stage 1: Identifying the research question

#### Objective

Main research question.

- What does diet and food consumption look like for Asian Americans in the general U.S. population?

Sub questions.

- What food groups and macronutrients are being consumed?
- What proportion and combinations of food groups are being consumed?
- What frequency are different food groups being consumed?
- How do diets differ across age groups (children, adolescent, adults, older adults)?
- How do diets differ across different Asian ethnic subgroups in the U.S.?
- What are the specific nutrition and dietary disparities?

#### Protocol and registration

To our knowledge, no previous review protocol or review exists for this question. We have registered our protocol on OSF (https://doi.org/10.17605/OSF.IO/K3X8S).

### Stage 2: Identifying the relevant studies that will be included in the scoping review

The review team includes a research librarian who developed a comprehensive search strategy. The search strategy combines four sets of terms:

1. Terms for diet, food, and nutrition
2. Terms for Asian American populations
3. Terms for eating behaviors, habits, and patterns
4. Terms for the United States

For each set of terms, the strategy uses the Boolean operator OR to combine database-specific structured vocabulary (when available) and free text keywords in the title/abstract field. The four sets of terms are combined with the Boolean operator AND. The search is limited to studies published since 2000 and studies published in English.

The core search strategy was developed in PubMed/MEDLINE and Nutrition and Food Science Collection (CABI Digital Library), to ensure that the search is sensitive for both population health literature and nutrition/food science literature. The strategy will be translated into additional databases for a comprehensive search: CINAHL (EBSCO), Scopus, Food Science and Technology Abstracts (Web of Science), and Biological & Agricultural Index Plus (EBSCO).

#### Core search strategy

*PubMed/MEDLINE*. (((diet* [title/abstract] OR food* [title/abstract] OR nutrition* [title/abstract] OR nutrient* [title/abstract] OR "diet, food, and nutrition" [mesh]) AND ("Asian American*" [title/abstract] OR "Bangladeshi American*" [title/abstract] OR "Bhutanese American*" [title/abstract] OR "Burmese American*" [title/abstract] OR "Cambodian American*" [title/abstract] OR "Chinese American*" [title/abstract] OR "Filipino American*" [title/abstract] OR "Hmong American*" [title/abstract] OR "Indian American*" [title/abstract] OR "Indo-Caribbean*" [title/abstract] OR "Indonesian American*" [title/abstract] OR "Japanese American*" [title/abstract] OR "Korean American*" [title/abstract] OR "Laotian American*" [title/abstract] OR "Malaysian American*" [title/abstract] OR "Mongolian American*" [title/abstract] OR "Pakistani American*" [title/abstract] OR "Taiwanese American*" [title/abstract] OR "Thai American*" [title/abstract] OR "Vietnamese American*" [title/abstract] OR "Asian American Native Hawaiian and Pacific Islander" [mesh])) AND ("behavior*" [title/abstract] OR "consumption" [title/abstract] OR "eating" [title/abstract] OR "habit*" [title/abstract] OR "intake" [title/abstract] OR "pattern*" [title/abstract] OR "eating" [mesh] OR "feeding behavior" [mesh])) AND ("United States" [title/abstract] OR "America*" [title/abstract] OR "U.S." [title/abstract] OR "United States" [mesh])

*Nutrition and Food Science Collection (CABI)*. "[[ab: diet*] OR [ab: food*] OR [ab: nutrition*] OR [ab: nutrient*] OR [de: diet] OR [de: nutrition]] AND [[ab: behavior*] OR [ab: consumption] OR [ab: eating] OR [ab: habit*] OR [ab: intake] OR [ab: pattern*] OR [de: "eating habits"] OR [de: "eating patterns"] OR [de: "consumption patterns"]] AND [[ab: america*] OR [ab: "u.s."] OR [ab: "united states"] OR [de: "united states of america"]] AND [[[ab: "asian american*"] AND [ab: "indo-caribbean*"]] OR [ab: "indonesian american*"] OR [ab: "japanese american*"] OR [ab: "korean american*"] OR [ab: "laotian american*"] OR [ab: "malaysian american*"] OR [ab: "mongolian american*"] OR [ab: "pakistani american*"] OR [ab: "taiwanese american*"] OR [ab: "thai american*"] OR [ab: "bangladeshi american*"] OR [ab: "vietnamese american*"] OR [ab: "bhutanese american*"] OR [ab: "burmese american*"] OR [ab: "cambodian american*"] OR [ab: "chinese american*"] OR [ab: "filipino american*"] OR [ab: "hmong american*"] OR [ab: "indian american*"]] AND [CABI Products: Nutrition and Food Science Collection]"

Additionally, hand searching will be conducted in the contents of all issues of the *Journal of Asian Health*, a journal not yet indexed in citation databases.

The review will also include a targeted search of grey literature to identify eligible studies not in the published literature. The grey literature search will search for dissertations in the ProQuest Dissertations & Theses Global database. We will also search reports, white papers, and resources released by U.S. Department of Agriculture (USDA) Food & Nutrition Service (FNS) and Asian American serving organizations, potentially including Asian American Federation, Asian & Pacific Islander American Health Forum, Asian American Research Center on Health, Asian American/Asian Research Institute City University of New York, Association of Asian Pacific Community Health Organizations, Center for the Study of Asian American Health, Coalition for Asian American Children and Families, Rutgers-NYU Center for Asian Health Promotion and Equity, and South Asian Public Health Association.

The search will be reported in accordance with the PRISMA-S guidelines [42].

### Stage 3: Selecting the studies

#### Eligibility (inclusion and exclusion) criteria

An article will be included if it

1. Was published in the English language
2. Is a peer-reviewed research manuscript from any discipline (articles with any study design, including reviews, systematic reviews, meta-analyses, commentaries, and editorials) or published in grey literature (dissertations, reports, white papers, and resources released by USDA FNS and Asian American serving organizations)
3. Explicitly focused on Asian Americans (either aggregated or by ethnic subgroups) in the general U.S. population
4. Includes healthy non-institutionalized populations of children, adolescents, adults, and/or older adults
5. Describes what food groups or macronutrients that healthy non-institutionalized Asian Americans living in the U.S. are or are not eating
6. Published in between 2000 to present

An article will be excluded if it

1. Did not have full text available
2. Had no explicit focus on the nutrition and dietary consumption of Asian Americans in the general U.S. population or if there is minimal description of diet
3. Only describes micronutrients that is being consumed
4. Contains only research conducted outside of the U.S.
5. Combines Asian Americans with Native Hawaiian and Pacific Islanders
6. Published before 2000
7. Describe neighborhood, community, environmental, social, and/or acculturation factors affecting diet but not does not describe food groups/macronutrients consumed

We will include articles with any study design including quantitative, qualitative, and mixed-methods studies. If more than one article originates from a study and report similar statistics or data, the most relevant and recent article will be chosen for review.

### Stage 4: Charting the data

#### Data charting process and data items

Before beginning the full review, the research team conducted a pilot of the title and abstract screening, full text review, and data extraction with the first 100 most recent results from PubMed to ensure the search strategy encompasses articles within the inclusion criteria and the data extraction form can capture information to answer the research questions.

The citations from each database will be imported into Covidence for de-duplication. Two reviewers will independently screen article titles and abstracts and full text for inclusion. Discrepancies will be resolved by a third reviewer. Data will be charted from the included articles using the data extraction form (Table 1) in Covidence by one reviewer. Data extracted included: article characteristics (journal, publication year, publication type, population focus), geographic focus, diet outcomes, and limitations and recommendations. Grey literature searches any relevant information will be separately recorded.

**Table 1:** Data extraction form.

**Basic Information**	
**Study/Covidence ID**	Free text
**Article title**	Free text
**Article first author** (e.g., First author last name et al. OR if only two authors, First & second author last names)	Free text
**Publication year**	Free text
**Journal**	Free text
**Reviewer name**	• Reviewer 1 • Review 2 • Reviewer 3
**Article type**	• Qualitative • Quantitative • Mixed methods • Review • Commentary • Editorial • Dissertation • Grey literature (specify below)
**If grey literature, please specify** (Report, white paper, infographic etc.)	Free text
**Age group** (multiple select)	• Children 0–12 • Adolescents 13–17 • Youths 0–17 • Young adults 18–24 • Adults 25–44 • Middle-aged adults 45–64 • Older adults 64+
**Asian group** (multiple select)	• Aggregated Asian American • Asian Indian • Bangladeshi • Bhutanese • Burmese • Chinese • Cambodian • Filipino • Hmong • Japanese • Laotian • Korean • Malaysian • Mongolian • Nepali • Pakistani • Sri Lankan • Thai • Vietnamese • Other (specify below)
**If other Asian group, please specify**	Free text
**Geographic location** (National or region or state)	Free text
**Language data collected in** (English or other Asian languages)	Free text
**Socioeconomic Status level** (multiple select)	• High • Middle • Low • Did not specify
**Dataset/Study name**	• Dataset 1 • Dataset 2 • Dataset 3
**Food Groups and Macronutrients Outcomes**	
**Calories**	Free text
List key conclusions/findings, comparisons, differences	
If none, write N/A	
**Carbohydrates**	Free text
List key conclusions/findings, comparisons, differences	
If none, write N/A	
**Protein** (includes meat, poultry, eggs, seafood, soy etc.)	Free text
List key conclusions/findings, comparisons, differences	
If none, write N/A	
**Oils & Fats**	Free text
List key conclusions/findings, comparisons, differences	
If none, write N/A	
**Vegetables**	Free text
List key conclusions/findings, comparisons, differences	
If none, write N/A	
**Fruits**	Free text
List key conclusions/findings, comparisons, differences	
If none, write N/A	
**Grains** (includes whole grains and refined grains)	Free text
List key conclusions/findings, comparisons, differences	
If none, write N/A	
**Dairy** (includes milk, yogurt, cheese, etc.)	Free text
List key conclusions/findings, comparisons, differences	
If none, write N/A	
**Added Sugars**	Free text
List key conclusions/findings, comparisons, differences	
If none, write N/A	
**Other Outcomes**	
**Food behaviors** (E.g. frequency of meals, location of meals, how food is eaten, where food is sourced from)	Free text
List key conclusions/findings, comparisons, differences	
If none, write N/A	
**Nutrition and dietary disparities observed across racial/ethnic groups**	Free text
List outcome, whether higher/lower/same for Asians (or ethnic group) compared to other ethnic/racial groups, other notes.	
If none, write N/A	
**Limitations & Recommendations**	
**Limitations mentioned specific for Asian American sample** (E.g. Asian American group not disaggregated, small sample, data collected only in English)	Free text
If none, write N/A	
**Recommendations mentioned for specific Asian American sample** (E.g. increase nutrition education, culturally appropriate interventions)	Free text
If none, write N/A	

### Stage 5: Collating, summarizing, and reporting the results

The conduct of the scoping review will be reported in accordance with the PRISMA-ScR checklist [43]. The screening and study selection process will be documented in a PRISMA-2020 flow diagram, including searches of databases and other sources. Key findings synthesizing recorded information will be summarized in narrative form. The manuscript will summarize results in relation to the main research question and the sub-questions organized by key themes, identify literature gaps, and describe implications for future research.

#### Patient and public involvement statement

Patients and the public will not be involved in the design, analysis, or reporting of this study. This study will solely synthesize information from publicly available publications.

#### Ethics and dissemination

Ethical approval is not required as we are not collecting and analyzing primary data. We are reviewing and extracting information from previously published studies and will disseminate study results through peer-reviewed publications.

## Discussion

Healthy dietary intake and patterns is important to address the rising chronic disease burden in the U.S. Mounting evidence indicates the longer-term vulnerability of Asian American populations who experience elevated risk and increased burden of cardiometabolic diseases [6–13]. Asian American populations face unique dietary challenges upon immigration to the U.S. that has been described as an explanatory factor in increased cardiometabolic risk and cancer burden in immigrant communities [19, 30, 31]. On top of existing structural inequities, Asian Americans also faced increased challenges in accessing food during the COVID-19 pandemic [44, 45], which can serve to exacerbate existing health disparities and emphasizes the need for policies and practices to address this issue in Asian American groups.

The aim of this review is to comprehensively summarize and synthesize the available evidence and knowledge on nutrition and dietary consumption in Asian American populations. We will summarize the available literature on the dietary intake and patterns among the general population organized by Asian American ethnic subgroup and highlight any disparities between groups. The findings from this review can have broad implications for designing and implementing nutrition-focused initiatives that will appropriately reflect and address the needs as well as norms and values of each distinct Asian American ethnic subgroup. Better and more nuanced understanding of different Asian American ethnic subgroups’ nutrition and dietary consumption can reveal practice implications and create policy opportunities to address health disparities in these communities. We plan to disseminate the results through a peer-reviewed manuscript.

## Supporting information

S1 File PRISM-P checklist. (DOCX)
